# The TvLEGU-1, a Legumain-Like Cysteine Proteinase, Plays a Key Role in *Trichomonas vaginalis* Cytoadherence

**DOI:** 10.1155/2013/561979

**Published:** 2013-01-01

**Authors:** Francisco Javier Rendón-Gandarilla, Lucero de los Angeles Ramón-Luing, Jaime Ortega-López, Ivone Rosa de Andrade, Marlene Benchimol, Rossana Arroyo

**Affiliations:** ^1^Departamento de Infectómica y Patogénesis Molecular, Centro de Investigación y de Estudios Avanzados del Instituto Politécnico Nacional, Avenida IPN No. 2508, Col. San Pedro Zacatenco, 07360 Mexico City, DF, Mexico; ^2^Departamento de Biotecnología y Bioingeniería, Centro de Investigación y de Estudios Avanzados del Instituto Politécnico Nacional, Avenida IPN No. 2508, Col. San Pedro Zacatenco, 07360 Mexico City, DF, Mexico; ^3^Laboratório de Ultraestrutura Celular, Universidade Santa Úrsula, Rua Jornalista Orlando Dantas 36, Botafogo, 22231-010 Rio de Janeiro, RJ, Brazil

## Abstract

The goal of this paper was to characterize a *Trichomonas vaginalis* cysteine proteinase (CP) legumain-1 (TvLEGU-1) and determine its potential role as a virulence factor during *T. vaginalis* infection. A 30-kDa band, which migrates in three protein spots (pI~6.3, ~6.5, and ~6.7) with a different type and level of phosphorylation, was identified as TvLEGU-1 by one- and two-dimensional Western blot (WB) assays, using a protease-rich trichomonad extract and polyclonal antibodies produced against the recombinant TvLEGU-1 (anti-TvLEGU-1r). Its identification was confirmed by mass spectrometry. Immunofluorescence, cell binding, and WB assays showed that TvLEGU-1 is upregulated by iron at the protein level, localized on the trichomonad surface and in lysosomes and Golgi complex, bound to the surface of HeLa cells, and was found in vaginal secretions. Additionally, the IgG and Fab fractions of the anti-TvLEGU-1r antibody inhibited trichomonal cytoadherence up to 45%. Moreover, the Aza-Peptidyl Michael Acceptor that inhibited legumain proteolytic activity in live parasites also reduced levels of trichomonal cytoadherence up to 80%. In conclusion, our data show that the proteolytic activity of TvLEGU-1 is necessary for trichomonal adherence. Thus, TvLEGU-1 is a novel virulence factor upregulated by iron. This is the first report that a legumain-like CP plays a role in a pathogen cytoadherence.

## 1. Introduction

Trichomoniasis is one of the most common sexually transmitted infections worldwide caused by *Trichomonas vaginalis *[1]. Trichomonal adherence to host cells is a multifactorial process where adhesins and proteinases play important roles [2–8]. Proteinases are abundant in *T. vaginalis, *being reported more than 400 distinct proteinase genes in the draft of its genome. Up to 220 correspond to the cysteine type (CP) [9], but only 23 CPs have been detected by two-dimensional (2D) substrate gel electrophoresis [10], less were identified by recent proteomic studies [11–15], and only few CP genes have been cloned and characterized [15–22]. These gene products show homology to cathepsin L-like peptidases, which belong to the papain-like CP family of clan CA and to the legumain-like CP family of clan CD [23, 24]. 

The thiol proteinases of this parasitic protozoan have been implicated in a variety of biological events including nutrient acquisition [25], immune evasion [26, 27], and virulence [1, 4, 5, 7, 8, 22, 28–35]. The expression of some of these CPs is regulated by environmental factors such as pH and the redox state [1], polyamines [31], and iron [18, 20, 21, 30, 32, 35]. 

Iron is an essential nutrient for growth, metabolism, and virulence of *T. vaginalis* [36]. The environment of the human vagina, especially its nutrients and the iron concentration, is constantly changing throughout the menstrual cycle. *T. vaginalis* may respond to varying iron concentrations by differential gene expression through poorly understood mechanisms [20, 21, 37] in order to survive, grow, and colonize the vaginal hostile environment.

We previously reported that some of the CPs of the 30-kDa region are involved in cytoadherence [4, 5, 7]. This region is formed by at least six spots with proteolytic activity that correspond to two distinct CP families: the papain-like family of clan CA, represented by four spots with pI between 4.5 and 5.5, and the legumain-like family of clan CD, represented by two spots with pI 6.3 and 6.5 [38] that are differentially regulated by iron at the transcript and proteolytic activity levels [21]. 

Among the ten legumain-like CP genes reported in the draft of the *T. vaginalis* genome [9], we have cloned and sequenced two cDNAs coding for the TvLEGU-1 and TvLEGU-2 precursor proteinases of 42.8- and 47.2-kDa. These CPs were classified within the asparaginyl endopeptidase (AE) subfamily of the family C13, belonging to the clan CD [38]. The family C13 of peptidases includes two distinct subfamilies with different functions, the glycosylphosphatidylinositol (GPI): protein transamidase and the asparaginyl endopeptidase. Interestingly, TvLEGU-1 and TvLEGU-2 share ~30% amino acid identity with the AE subfamily and ~26% with the GPI: protein transamidase subfamily [38]. We also showed that the amount of TvLEGU-1 transcript is positively regulated by iron, whereas the TvLEGU-2 mRNA is not affected by it [21]. Additionally, TvLEGU-1 is one of the most immunogenic proteinases detected by trichomoniasis patient sera [15].

Thus, the main goal of this work was to identify, characterize, and determine the function of TvLEGU-1. Our data show that TvLEGU-1 is a surface proteinase upregulated by iron, with affinity to the surface of HeLa cells that plays a major role in trichomonal cytoadherence. Hence, TvLEGU-1 is a novel virulence factor of *T. vaginalis* that is also released in vaginal secretions during infection.

## 2. Materials and Methods

### 2.1. Parasites and HeLa Cell Cultures

The fresh clinical *T. vaginalis* isolate CNCD 147 [7, 15, 29] was used in this study. Parasites were kept in culture at 37°C up to two weeks by daily passage in trypticase-yeast extract-maltose (TYM) medium [39] supplemented with 10% heat-inactivated horse serum (HIHS) (TYM-HIHS), containing ~20 *μ*M iron [36]. Parasites in the logarithmic phase were grown either in iron-rich or in iron-depleted medium by the addition into the culture medium of 250 *μ*M ferrous ammonium sulfate or 150 *μ*M 2-2 dipyridyl (Sigma Co., St Louis, MO, USA) an iron-chelator, respectively, as previously reported [30]. HeLa cells were grown in Dulbecco's Modified Eagle Medium (DMEM) (Gibco Laboratories, Grand Island, NY) supplemented with 10% HIHS at 37°C for 48 h in a 5% CO_2_ atmosphere to obtain confluent cell monolayers [6].

### 2.2. Generation of Antiserum against Recombinant TvLEGU-1

Rabbits were subcutaneously inoculated four times at two-week intervals with 0.3 mg of the affinity-purified TvLEGU-1r protein [15] in the presence of Freund's complete adjuvant (Gibco) for the first immunization. Booster injections were given in Freund's incomplete adjuvant (Gibco). The immune serum (anti-TvLEGU-1r) was obtained seven days after the last immunization [40]. This antiserum was used in western blot (WB) analysis, indirect immunofluorescence, and cytoadherence inhibition assays. Preimmune (PI) serum was obtained before the immunization schedule began and was used as a negative control in all the experiments with antibodies.

### 2.3. Papain Fragmentation of IgG to Fab

To obtain the Fab fragment, 0.5 mg/mL purified IgGs from the anti-TvLEGU-1r or PI serum [40, 41] in PBS pH 8.0 were digested with 0.2 mg/mL papain in digestion buffer (PBS pH 8.0 containing 0.02 M cysteine and 0.02 M EDTA) at 37°C for 6 h. The reaction was stopped with 0.3 M iodoacetamide in PBS pH 8.0. After digestion, samples were dialyzed in PBS pH 7.0 for 18 h at 4°C and incubated with protein A agarose during 2 h to eliminate the Fc fraction and recover the unbound Fab fragment [40].

### 2.4. Two-Dimensional Gel Electrophoresis (2DE)

The 2DE for protease-rich extracts was performed as recently described [22]. Briefly, for the first dimension, supernatant from lysed parasites (6 × 10^7^ cells/mL equivalent to 500 **μ**g protein) in rehydration solution (Bio-Rad) was loaded onto a 7 cm Ready immobilized pH gradient (IPG) strips (linear pH gradient 4–7; Bio-Rad). IPG strips were actively rehydrated for 16 h at 4°C. Isoelectric focusing (IEF) of proteins was performed in three steps: 250 V for 20 min, 4 000 V for 3 h, and a gradual increase up to 10 000 V-h. For reduction and alkylation, strips were equilibrated in buffer I and II (Bio-Rad) for 10 min at room temperature each. Proteins were resolved by SDS-PAGE using 12% polyacrylamide gels, silver-stained, or transferred onto nitrocellulose (NC) membrane for WB detection. Gels and NC membranes were documented using the ChemiDoc-XRS (Bio-Rad) and analyzed using the Quantity One software (Bio-Rad). A tridimensional analysis using the PD Quest (Bio-Rad) and Melanie software was also performed for differentially expressed proteins. Three independent protein preparations were done, each obtained from an independent parasite culture, and similar results were observed.

### 2.5. Proteinase Identification

Identification of protein spots was performed at the Protein Unit of the Columbia University (NY, USA) as before [15]. Protein spots of interest were manually excised from silver-stained gels, distained, and prepared for in-gel digestion with trypsin. Resulting peptides were analyzed by MALDI-TOF mass spectrometry (MS) peptide mass mapping method on a Voyager DE pro-mass spectrometer in the linear mode (Applied Biosystems). Peptide masses were searched against the National Center for Biotechnology nonredundant database (NCBInr) using the MASCOT program (http://matrixscience.com/).

### 2.6. Western Blot Analysis (WB)

Total trichomonad proteins, proteinase-rich extracts, proteins obtained after cell-binding assays from (2 × 10^7^) parasites grown in iron-rich medium, obtained as before [7], and TCA-precipitated proteins present in vaginal washes (VWs) (100 **μ**L) from patients with vaginitis (Table 2) were separated by SDS-PAGE using 10% polyacrylamide gels. Duplicated gels were transferred onto NC membranes for WB. The TvLEGU-1 proteinase was immunodetected with the anti-TvLEGU-1r rabbit serum (at 1 : 1000 to 1 : 40000 dilutions). As a quantity control, a monoclonal antibody against *α*-tubulin (Zymed Laboratories, South San Francisco, CA) (at 1 : 100 dilution) was used. WB was developed by chemiluminescence using the ECL-Plus kit (Amersham Co., Arlington Heights, IL, USA) and SuperSignal Femto Maximum Sensitivity Substrate kit (Pierce) and documented using the ChemiDoc-XRS (Bio-Rad). 

To determine the presence of phosphorylations on TvLEGU-1, anti-phospho-Ser, -Tyr, and -Thr monoclonal antibodies at 1 : 500 dilution (Zymed) were used in 2DE-WB assays over NC membranes containing protease-rich extracts and developed by chemiluminescence. These experiments were performed at least three times with similar results.

### 2.7. *In Vitro* Secretion Kinetic Assay

The *in vitro* secretion assay was performed as previously described [22, 33]. Briefly, after 18 h of growth in iron-rich conditions, parasites were harvested, washed three times with PBS pH 7.0, and suspended in PBS-0.5% maltose at 1 × 10^6^ cells/mL parasite density. Parasites were incubated for 15, 30, 60, and 90 min at 37°C, collected by centrifugation at 700 g, and supernatants were analyzed directly by substrate-gel electrophoresis and by WB after TCA-precipitation. The viability of trichomonads was assessed by trypan blue exclusion throughout the assay. 

### 2.8. Indirect Immunofluorescence Assay

For confocal microscopy, parasites grown in iron-rich conditions were fixed with 4% paraformaldehyde for 1 h at 37°C, washed with PBS, and half of them were treated with 50 mM NH_4_Cl/PBS pH 7.0 for 10 min, washed with PBS, and with 1 N HCL for 1 h and permeabilized with 0.2% Triton X-100 for 10 min. The other half was used as nonpermeabilized parasites. Permeabilized and nonpermeabilized parasites were blocked with 1% fetal bovine serum for 15 min and with 0.2 M glycine for 1 h at room temperature. Then, trichomonads were incubated for 18 h at 4°C, with the anti-TvLEGU-1r or PI serum used as a negative control, both at 1 : 1 000 dilution. Parasites were incubated with the secondary antibody, fluorescein isothiocyanate-conjugated anti-rabbit immunoglobulins (Pierce) at 1 : 200 dilution for 1 h at 37°C, washed, mounted with Vectashield mounting solution (Vector Laboratories), and visualized by confocal microscopy with a Leica LSM-SPC-5 Mo inverted confocal microscope fitted with HCXPLapo lambda blue 63 × 1.4 oil immersion lens. Time series were captured and processed using the confocal LAS AF software (Leica). Also, live HeLa cells were incubated with 10 **μ**g/mL TvLEGU-1r or supernatant from an *in vitro *secretion assay for 30 min at 37°C, washed with PBS, fixed, blocked, and treated with antibodies as the parasites described above for immunofluorescence assays. 

For lysosomal colocalization assays, the acidic compartments of *T. vaginalis* were stained with 1 **μ**M LysoTracker RED DND-99 (Invitrogen) for 12 h at 37°C in TYM medium supplemented with 10% heat-inactivated horse serum. After that, parasites were processed for indirect immunofluorescence with the anti-TvLEGU-1r antibody as described in the previous paragraph. 

For immunogold labeling assays, parasites were fixed overnight at room temperature in 0.5% glutaraldehyde, 4% formaldehyde in 0.1 M cacodylate buffer. Afterwards, cells were dehydrated in ethanol and embedded in Unicryl. Ultra-thin sections were harvested on 300 mesh nickel grids. The samples were washed and incubated with 50 mM ammonium chloride for 30 min in order to quench free aldehyde groups. The sections were incubated in a series of blocking solutions (PBS containing 1% bovine albumin (BSA), 3% PBS/BSA, and 0.2% Tween-20, pH 8.0) for 10 min on each step. Cells were incubated with the anti-TvLEGU-1r antibody at 1 : 50 dilution, overnight. After several washes in 1% PBS/BSA, the sections were incubated with 10 nm gold-labeled goat anti-rabbit IgG (BB International, UK). As control some samples were incubated only with the secondary antibody. Finally, sections were stained with 5% uranyl acetate and 1% lead citrate and then observed with a JEOL 1210 transmission electron microscope.

### 2.9. Cell-Binding Assay for Proteinases

To detect the affinity of the native and recombinant TvLEGU-1 proteins to the surface of host cells, we performed cell-binding assays as previously described [7]. Briefly, a clarified detergent extract from 2 × 10^7^ parasites or 25 **μ**g of TvLEGU-1r was incubated for 18 h at 4°C with 1 × 10^6^ glutaraldehyde-fixed HeLa cells. The native and recombinant TvLEGU-1 proteins bound to the surface of fixed-HeLa cells were eluted with Laemmli sample buffer for 20 min at 37°C. The released proteins were analyzed by SDS-PAGE and blotted onto NC for WB detection with the anti-TvLEGU-1r antibody.

### 2.10. Cytoadherence Inhibition Assay

Cytoadherence inhibition assays were performed over confluent HeLa cell monolayers on 96-well microtiter plates as previously described [7, 42]. Briefly, [^3^H]-thymidine-labeled parasites were incubated for 30 min at 4°C with 0, 50, and 100 **μ**g/mL IgG or Fab fraction from the anti-TvLEGU-1r or PI serum before interaction with HeLa cell monolayers. 

The cytoadherence inhibition assays with proteinase inhibitors were performed over confluent live HeLa cell monolayers on 12 mm coverslips as recently described [41]. Briefly, cell monolayers (5 × 10^5^ cells/coverslip) were incubated with live parasites (1 × 10^6^ cells/well) previously labeled with 25 mM CellTracker Blue CMAC (Molecular Probes) in serum-free DMEM-TYM (2 : 1) medium and incubated at 37°C for 30 min and 5%  CO_2_. For inhibition experiments before interaction with HeLa cell monolayers labeled parasites were incubated for 20 min at 4°C with different CP inhibitors (1 mM TLCK, 0.2 mM leupeptin, or 0.18 mM E-64; all purchased from Sigma) used as controls. The Aza-Peptidyl Michael Acceptor (Mu-Ala-Ala-AAsn-CH=CH–CON, kindly donated by Dr. James Powers), a specific inhibitor for legumains [43], was also used at 5, 10, and 50 **μ**M. After interaction with CP inhibitors parasites were washed and added to HeLa cell monolayers. After the interaction, the coverslips were washed with warm PBS, fixed with 4% paraformaldehyde, and mounted on slides. Each condition was performed in triplicate, and ten fields with a 40x magnification were analyzed per coverslip. Fluorescent parasites adhered to host cells (in blue) were counted using an Eclipse 80i epifluorescence microscope (Nikon) and the NIS-Elements BR 2.1 software (Nikon) (Table 3). The experiment was repeated at least two independent times with similar results. 

### 2.11. Measurement of the Proteolytic Activity of Live Parasites

To detect the proteolytic activity of live trichomonads, 2.5 × 10^5^ parasites were incubated for 20 min at 4°C with 1 mM TLCK, 0.2 mM leupeptin, 0.18 mM E-64, or 50 **μ**M Aza-Peptidyl Michael Acceptor [43]. The activity was measured with two CPs substrates (Z-Phe-Arg-AMC for papains; and Cbz-Ala-Ala-AAsn-AMC for legumains). Release of free 7-amino-4-methylcoumarin (AMC) was measured by emission at excitation wavelengths of 355 and 460 nm, respectively, in a luminometer (BioTek) using a Gen5 2.0 Data Analysis Software. The linear regression of the substrate hydrolysis curves was used to calculate initial velocities. The experiment was repeated at least three independent times with similar results. The viability of trichomonads was assessed by trypan blue exclusion throughout the assay.

### 2.12. Statistical Analysis

The statistically significant difference between means was determined by analysis of variance (ANOVA) using GraphPad Prism 5.0. The data were analyzed by one-way ANOVA using the Bonferroni method comparing all pairs of columns (*P* < 0.001) for Figures 7(A), 7(B), 8(B), 8(C), and 8(D). The scores showing statistical significance are indicated in the figures with asterisks. The corresponding *P* values are indicated in the figure legends.

## 3. Results

### 3.1. Identification of the TvLEGU-1 Proteinase

**Figure 1:**
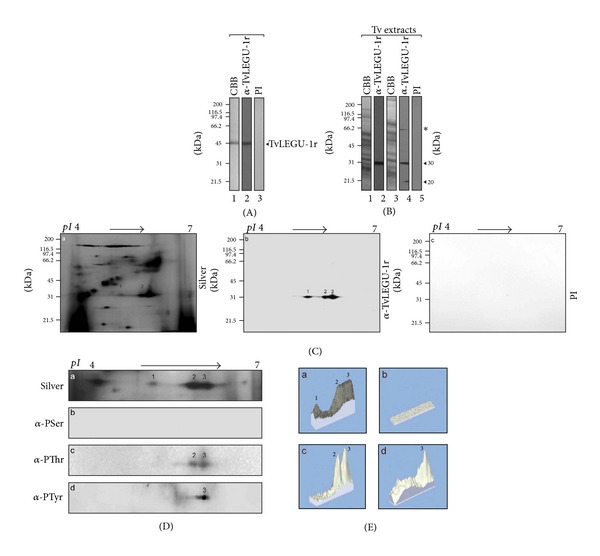
Recognition of TvLEGU-1 by the anti-TvLEGU-1r antibody in total protein extracts and protease-rich extracts of *T. vaginalis*. (A) Coomassie brilliant blue-stained purified recombinant TvLEGU-1r protein (CBB; lane 1). WB assays of TvLEGU-1r incubated with the anti-TvLEGU-1r antibody (lane 2) or with the PI serum (lane 3) used as a negative control, both at 1 : 40000 dilution. Arrowhead points to the recombinant protein band TvLEGU-1r (~46-kDa). kDa, molecular weight markers in kilodaltons (Bio-Rad). (B) Coomassie brilliant blue-stained protein patterns of trichomonad protease-rich (lane 1) or total protein extracts (lane 3); WB assays of duplicated gels transferred onto NC membranes containing trichomonad protease-rich (lane 2) or total protein extracts (lane 4) incubated with the anti-TvLEGU-1r antibody, or with the PI serum (lane 5) used as a negative control, both at 1 : 20000 dilution. Arrowheads show the position of the native TvLEGU-1 (~30- and ~20-kDa) proteins. Asterisk shows a light protein band of ~60-kDa also detected by the anti-TvLEGU-1r antibody. kDa, molecular weight markers in kilodaltons (Bio-Rad). (C) Silver-stained 2DE protease-rich extracts from parasites grown in regular medium (panel a). WB of duplicate gels transferred onto NC membranes incubated with the anti-TvLEGU-1r antibody (panel b) or with the PI (panel c) serum both at 1 : 5000 dilution. Numbers 1–3 show the position of the TvLEGU-1 protein spots. (D) Differential phosphorylation of TvLEGU-1. 2DE WB assays from duplicate protease-rich extracts from part (C) separated by SDS-PAGE were (a) silver-stained or transferred onto NC membranes and incubated with the (b) antiphosphoserine (*α*-PSer), (c) antiphosphothreonine (*α*-PThr), and (d) antiphosphotyrosine (*α*-PTyr) monoclonal antibodies. pI, direction of IEF using IPG Ready-strips (linear gradient of pH 4–7; Bio-Rad). Numbers show the position of the TvLEGU-1 spots. (E) Landscape representation of the densitometric analysis (PDQuest and Melanie software) of the protein spots corresponding to TvLEGU-1. Numbers 1–3 show the position of the TvLEGU-1 spots.

To identify the TvLEGU-1 CP in *T. vaginalis* proteinase-rich extracts polyclonal antibodies were produced against the recombinant TvLEGU-1 protein (anti-TvLEGU-1r) previously cloned and expressed in *Escherichia coli *[15]. By WB assays, the anti-TvLEGU-1r antibody reacted with the recombinant TvLEGU-1 protein used as antigen (Figure 1(A)). It also recognized a 30-kDa band in *T. vaginalis* protease-rich trichomonad extracts and two bands of 30- and 20-kDa in *T. vaginalis* total protein extracts of parasites grown in normal iron conditions. A light band of 60-kDa was also observed (Figure 1(B)). As expected, the PI serum used as a negative control had no reaction (Figure 1).

To confirm the identity of the protein spots corresponding to TvLEGU-1 in a protease-rich trichomonad extract (dubbed “active degradome”) [15], parasites grown in normal iron conditions were analyzed by 2DE and WB assays. The anti-TvLEGU-1r antibody recognized three spots in the 30-kDa region with pI ~6.3, ~6.5, and ~6.7 (Figure 1(C)). These protein spots were identified by MALDI-TOF MS analysis as TvLEGU-1 proteins; ten of the 25 peptides obtained by tryptic digestion of the three spots had identical masses to TvLEGU-1 peptides. The MS analysis showed that protein spots 1, 2, and 3 were identified with MASCOT scores of 72, 85, and 150, respectively, and sequence coverage of 14, 33, and 31%, respectively; reinforcing their identification as part of the TvLEGU-1 protein (Figure 1; Table 1; Supplementary Figure  1S). 

### 3.2. TvLEGU-1 Is Phosphorylated and Upregulated by Iron

To investigate whether phosphorylation could be an explanation for the three protein spots with the same size but distinct pI identified as TvLEGU-1 in 2DE WB assays, protease-rich extracts from parasites grown in normal iron concentrations were analyzed by 2DE WB assays with antiphospho-Ser, -Thr, and -Tyr antibodies. Figure 1(D) shows that while none of the protein spots have phosphorylation in Ser residues, the protein spot 1 did not show either in Thr or Tyr residues, the protein spots 2 and 3 showed in Thr, and only spot 3 in Tyr residues. Interestingly, the intensity of the protein spot 3 was greater with the anti-Tyr than with the anti-Thr antibody (Figures 1(D) and 1(E)). Thus, the three protein spots of TvLEGU-1 are isoforms with distinct type and degree of phosphorylation, as was predicted in its amino acid sequence [38].

**Figure 2:**
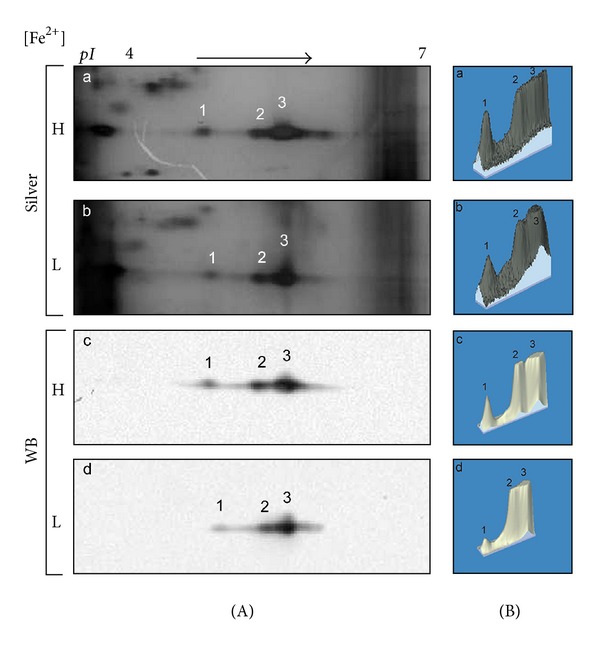
Iron effect on the protein expression of TvLEGU-1. (A) Silver-stained 2DE pattern of protease-rich extracts, corresponding to the 30-kDa region from (a) parasites grown in iron-rich (H) or (b) iron-depleted (L) conditions. (c) and (d) 2DE WB assays of duplicated gels (a) and (b) transferred onto NC membranes and incubated with the anti-TvLEGU-1r antibody (1 : 5000 dilution). pI, direction of IEF using IPG Ready-strips (linear gradient of pH 4–7; Bio-Rad). Numbers 1–3 show the position of the distinct TvLEGU-1 protein spots. (B) Landscape representation of the densitometric analysis of the three TvLEGU-1 protein spots of (a) and (b) silver-stained gels and (c) and (d) WB shown in (A) was carried out with the Melanie and PDQuest (Bio-Rad) programs. Numbers 1–3 correspond to the protein spots of TvLEGU-1.

**Figure 3:**
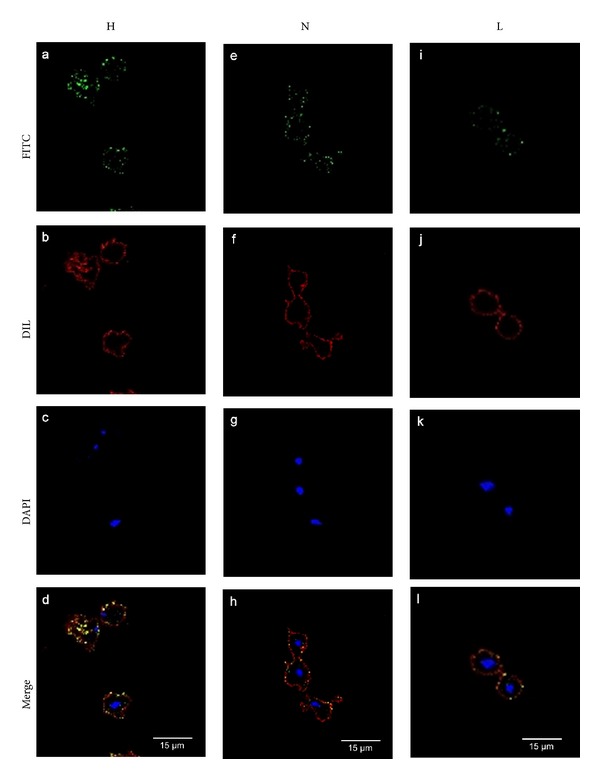
The TvLEGU-1 surface localization on *T. vaginalis *is affected by iron. Parasites grown in iron-rich (H; a, b, c, and d), normal (N; e, f, g, and h), and iron-depleted (L; i, j, k, and l) conditions, fixed and nonpermeabilized were incubated with the anti-TvLEGU-1r antibody (1 : 100 dilution). Anti-rabbit IgG-FITC (in green) was used as a secondary antibody (1 : 100 dilution) (a, e, and i). Parasite membranes were labeled with DIL (in red; b, f, and j). Nuclei were labeled with DAPI (in blue; c, g, and k). Merge (d, h, and l) in yellow indicates colocalization. Bars: 15 *μ*m (d, h, and l).

To check the effect of iron at the protein expression level of TvLEGU-1, protease-rich extracts from parasites grown in iron-rich and iron-depleted conditions were analyzed by silver-stained 2DE and by 2DE WB. A densitometric analysis was also performed (Table 2).  Figure 2 shows that the three protein spots in the 30-kDa region are present in both iron conditions but protein spot 1 with less intensity in iron-depleted than in iron-rich parasites (Figure 2(A)). These differences are well appreciated in the densitometric analysis (Figure 2(B), Table 2). Furthermore, the surface localization of TvLEGU-1 in nonpermeabilized parasites grown in different iron concentrations was explored. The data show that TvLEGU-1 surface localization is positively modulated by iron (Figure 3). Only a light surface localization of TvLEGU-1 was observed in iron-depleted parasites. These data together with the 2D WB results suggest that the major differences of this protein due to the iron concentration could be observed at its surface localization. Therefore, the rest of the experiments involving the anti-TvLEGU-1r antibody were performed with parasites grown in iron-rich conditions, except when indicated.

### 3.3. TvLEGU-1 Is Localized in the Cytoplasm and on the Surface of *T. vaginalis*

**Figure 4:**
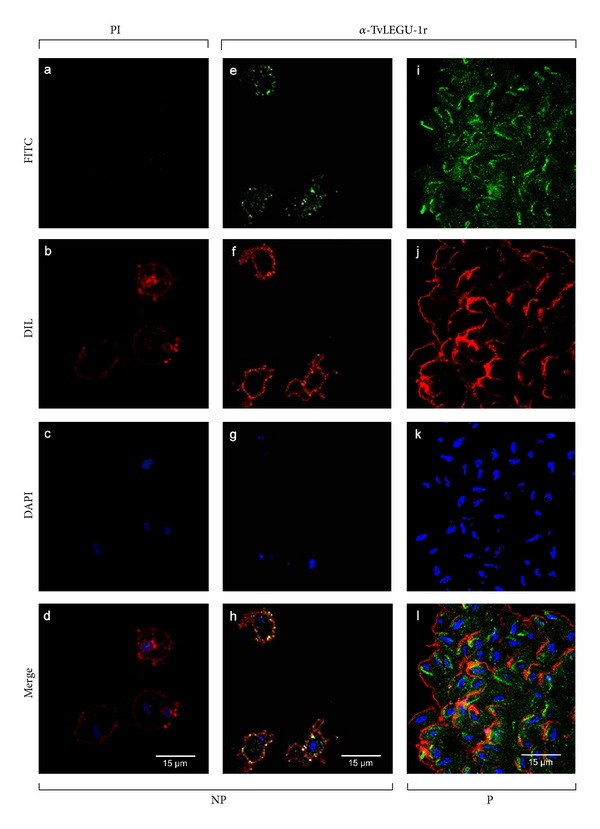
The TvLEGU-1 protein is localized on the surface and cytoplasm of *T. vaginalis*. Parasites grown in iron-rich conditions, fixed nonpermeabilized (NP) were incubated with the PI serum, (1 : 100 dilution) as a negative control (a, b, c, and d). Nonpermeabilized (NP; e, f, g, and h) and permeabilized (P; i, j, k, and l) parasites were incubated with the anti-TvLEGU-1r antibody (1 : 100 dilution). Anti-rabbit IgG-FITC (in green) was used as a secondary antibody (1 : 100 dilution) (a, e, and i). Parasite membranes were labeled with DIL (in red; b, f, and j). Nuclei were labeled with DAPI (in blue; c, g, and k). Merge (d, h, and l) in yellow indicates colocalization. Bars: 15 *μ*m (d, h, and l).

To explore the total real distribution of TvLEGU-1 in trichomonads immunofluorescence assays were performed using the anti-TvLEGU-1r antibody with fixed nonpermeabilized or permeabilized parasites grown in iron-rich condition and analyzed by confocal microscopy.  Figure 4 shows that fluorescence with the anti-TvLEGU-1r antibody (in green) was detected on the surface, colocalizing with the membrane marker (Dil, in red; panels e–h) and in the cytoplasm of trichomonad parasites (panels i–l) as compared with the PI serum used as a negative control (panels a–d). A very interesting labeling in the Golgi complex and vesicles that could be lysosomes was observed in the cytoplasmic localization of TvLEGU-1. 

**Figure 5:**
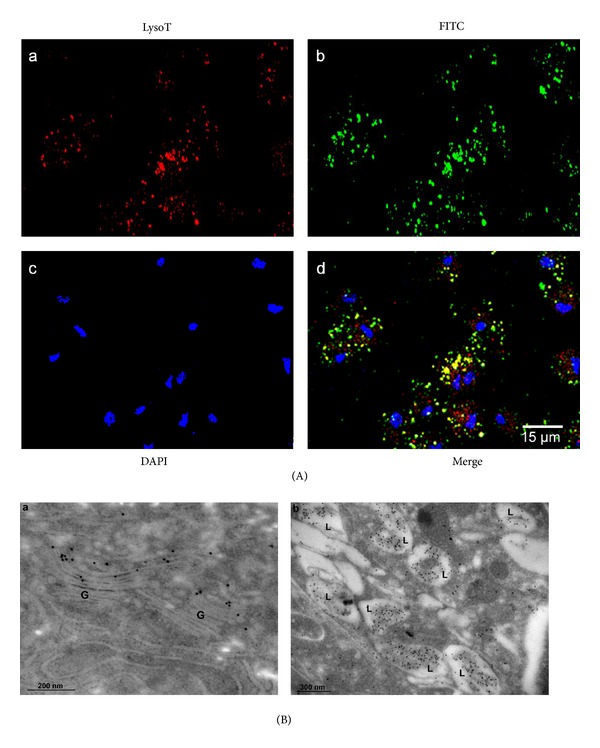
TvLEGU-1 is also localized in lysosomes and Golgi complex of *T. vaginalis.* (A) Parasites grown in iron-rich and labeled with LysoTracker were fixed, blocked, and incubated with the anti-TvLEGU-1r antibody (1 : 100 dilution) and a secondary antibody coupled to FITC stained in green; lysosomes were stained in red with LysoTracker, LysoT; and nuclei in blue with DAPI. The samples were analyzed by confocal microscopy. (B) Immunogold labeling of thin cryosections of parasites using the anti-TvLEGU-1r antibody at 1 : 100 dilution. The samples were analyzed by transmission electron microscopy. The labeling is observed on (a) Golgi complex (G) and (b) vesicles similar to lysosomes (L). Bars: 200 nm and 300 nm, respectively.

To explore the hypothesis that TvLEGU-1 is also a lysosomal CP, we performed colocalization assays using LysoTracker as a lysosomal marker in addition to the anti-TvLEGU-1r antibody.  Figure 5 shows that indeed TvLEGU-1 colocalized (~60%) with the lysosomal marker in iron-rich parasites, as could be expected for legumain-like CPs (Figure 5(A)). Moreover, immunogold localization assays confirmed the cytoplasmic localization of TvLEGU-1 in vacuoles/lysosomes containing degrading material and in the Golgi complex (Figure 5(B)), suggesting that this is an excreted/secreted proteinase. These data suggest that TvLEGU-1 could have multiple functions that will depend on its cellular localization possible modulated by the iron concentrations. 

### 3.4. TvLEGU-1 Binds to the Surface of HeLa Cells

**Figure 6:**
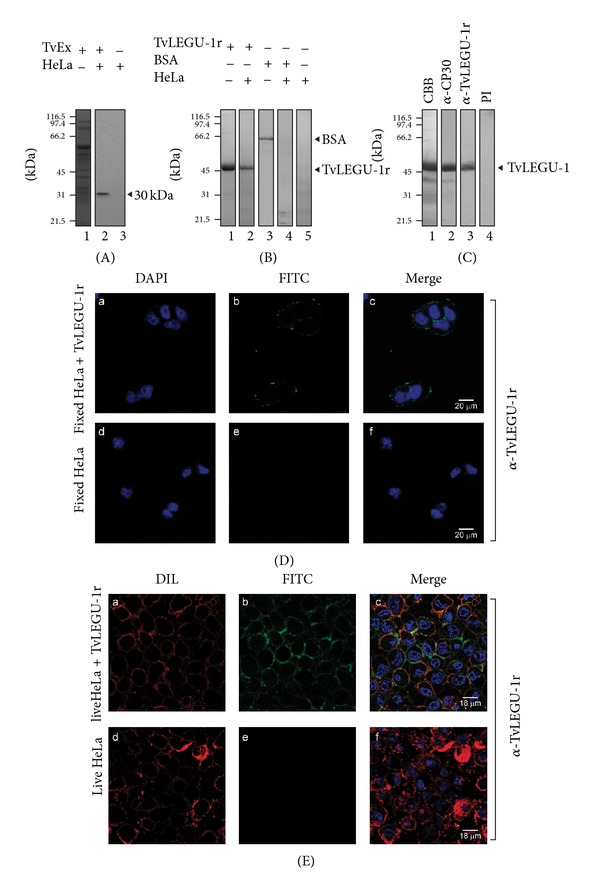
TvLEGU-1 binds to the surface of fixed and live HeLa cells. (A) Coomassie brilliant blue-stained protease-rich extracts from parasites grown in iron-rich conditions (lane 1). WB assay of eluted proteinases after cell-binding assay of protease-rich extracts with fixed HeLa cells (lane 2) or mock HeLa cells (lane 3) incubated with the anti-TvLEGU-1r antibody (1 : 1 000 dilution). (B) The TvLEGU-1r protein interacts with fixed HeLa cells. Coomassie brilliant blue-stained TvLEGU-1r protein (lane 1) and TvLEGU-1r protein eluted after cell-binding assays with fixed HeLa cells (lane 2). Coomassie brilliant blue-stained bovine serum albumin (BSA) (lane 3) and BSA eluted after cell-binding assays with fixed HeLa cells (lane 4) used as a specificity control; mock experiment with fixed HeLa cells (lane 5). (C) Recognition of TvLEGU-1r by the anti-CP30 antibody. Coomassie brilliant blue-stained TvLEGU-1r (lane 1); WB assay of the TvLEGU-1r protein incubated with the anti-CP30 (1 : 5 000 dilution) [7] or the anti-TvLEGU-1r antibody (1 : 10000 dilution), or PI serum (1 : 1 0000 dilution) used as a negative control (lanes 2, 3, and 4, resp.); kDa, molecular weight markers in kilodaltons. Protein bands were visualized by SDS-PAGE on 10% polyacrylamide gels. Arrowheads show the position of TvLEGU-1 (A), BSA (B), or TvLEGU-1r ((B) and (C)) proteins. (D) Confocal microscopy images after immunofluorescence assays using the anti-TvLEGU-1r antibody with fixed HeLa cells incubated with the TvLEGU-1r protein (a, b, and c). Fixed HeLa cells directly incubated with the anti-TvLEGU-1r antibody were used as negative controls. Conjugated anti-rabbit IgG-FITC was used as a secondary antibody (1 : 100 dilution) (b and e). Nuclei stained with DAPI (a and d). Merge and bars: 20 *μ*m (c and f). (E) Confocal microscopy images after immunofluorescence assays using the anti-TvLEGU-1r antibody with live HeLa cells incubated with the TvLEGU-1r protein (a, b, and c). Live HeLa cells were directly incubated with the anti-TvLEGU-1r antibody and used as negative controls (d, e, and f). Conjugated anti-rabbit IgG-FITC was used as a secondary antibody (1 : 100 dilution) (b and e). Parasite membranes were labeled with DIL (a and d). Nuclei labeled with DAPI, merge, and bars: 18 *μ*m (c and f).

To determine whether TvLEGU-1 binds to the surface of HeLa cells, cell-binding and WB assays were performed with protease-rich extracts from parasites grown in iron-rich medium and fixed HeLa cells. WB assays showed that the anti-TvLEGU-1r antibody reacted with the trichomonad 30-kDa band that bound to fixed HeLa cells (Figure 6(A)), suggesting the presence of TvLEGU-1. This was confirmed in a cell-binding assay using the recombinant TvLEGU-1 protein that also bound to the surface of fixed HeLa cells (Figure 6(B)), whereas the bovine serum albumin (BSA) used as a negative control did not bind as expected (Figure 6(B)). Additionally, TvLEGU-1r was recognized by the antinative CP30 antibody [7] in WB assays (Figure 6(C)). Together, these data show that TvLEGU-1 is one of the CP30 proteinases that interact with the surface of HeLa cells [7].

Furthermore, to confirm it, immunofluorescence assays using fixed and live HeLa cells incubated with the TvLEGU-1r protein and the anti-TvLEGU-1r antibody were performed. Confocal microscopy images showed that indeed TvLEGU-1r bound to the surface of fixed and live HeLa cells, whereas HeLa cells directly incubated with the anti-TvLEGU-1r antibody used as a negative control had no reaction as expected (Figures 6(D) and 6(E)). 

### 3.5. TvLEGU-1 Participates in *T. vaginalis* Cytoadherence

**Figure 7:**
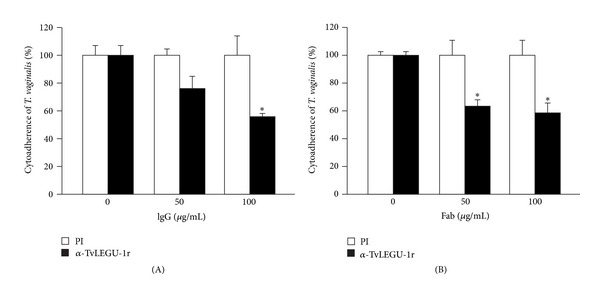
TvLEGU-1 participates in *T. vaginalis *cytoadherence. For cytoadherence inhibition experiments, [^3^H]-thymidine-labeled iron-rich parasites (1 × 10^6^ cell/mL) were incubated for 20 min at 4°C with different concentrations (0, 50, and 100 *μ*g/mL) of the IgG (A) and Fab (B) fractions from the anti-TvLEGU-1r or PI serum before interaction with HeLa cell monolayers. (A) Cytoadherence inhibition with IgG fractions from the anti-TvLEGU-1r (black bar) or PI (white bar) serum. (B) Cytoadherence inhibition with Fab fractions from the anti-TvLEGU-1r (black bar) or PI (white bar) serum. Each bar is the mean of the percentage of cytoadherence of triplicate samples; error bars represent the standard deviations of three experiments in triplicate with similar results. *P < 0.05 is the significance of the difference between 100 *μ*g/mL IgG fractions of the control PI serum and the anti-TvLEGU-1r serum. *P < 0.001 is the significance of the difference between 50 or 100 *μ*g/mL Fab fractions of the control PI serum and the anti- TvLEGU-1r serum.

To study the role of TvLEGU-1 in trichomonal adherence, we performed adherence inhibition assays over HeLa cell monolayers by preincubating [^3^H]-thymidine-labeled iron-rich parasites with varied concentrations of the anti-TvLEGU-1r IgG or Fab fractions.  Figure 7 shows that the anti-TvLEGU-1r antibody inhibited the levels of *T. vaginalis *adherence to HeLa cell monolayers in a concentration-dependent manner. A maximum inhibition of ~45%, using 100 **μ**g/mL of IgGs or Fab fractions, was observed. IgGs or Fab fractions from PI serum used as a negative control did not affect trichomonal cytoadherence. These results illustrate that TvLEGU-1 is a virulence factor that plays a role in cellular attachment as one of the 30-kDa CPs required for trichomonal adherence [7].

### 3.6. TvLEGU-1 Proteolytic Activity Is Necessary for *T. vaginalis* Cytoadherence

**Figure 8:**
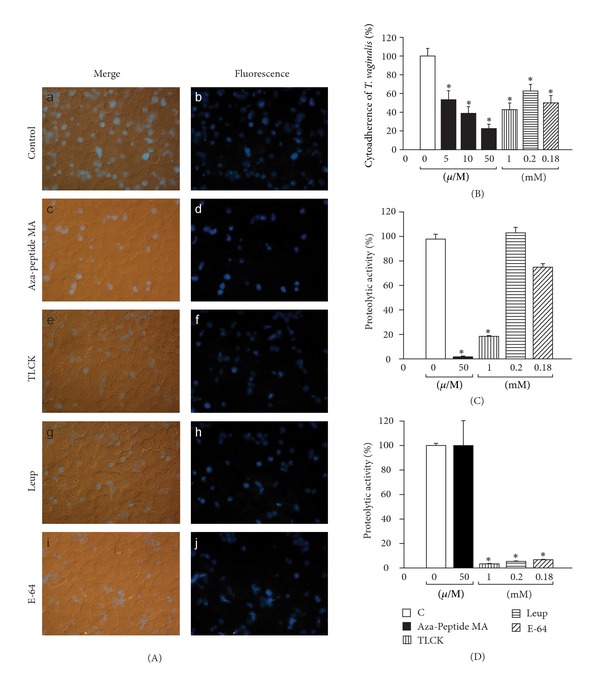
Effect of distinct CP inhibitors in trichomonal cytoadherence ((A) and (B)) and proteolytic activity of live parasites ((C) and (D)). (A) Fluorescence microscopy of a representative cytoadherence inhibition assay (over live HeLa cell monolayers) of fluorescence-labeled parasites pretreated with different CP inhibitors. Panels a and b show parasites without treatment (100% adherence). Panels c and d correspond to parasites treated with 50 *μ*M legumain inhibitor Aza-Peptide Michael Acceptor (Mu-Ala-Ala-AAsn-CH=CH-CON). Panels e and f show parasites treated with 1 mM TLCK. Panels g and h, parasites treated with 0.2 mM Leupeptin (Leup). Panels i and j, parasites treated with 0.18 mM E-64. (B) Data from the fluorescent parasites of the cytoadherence inhibition assay show the percentage of *T. vaginalis* bound to HeLa cell monolayers in the absence (0, used as a control) or presence of distinct concentrations of CP inhibitors described in (A). Each bar is the mean of the percentage of triplicate samples; error bars represent the standard deviations of two experiments in triplicate with similar results. *P < 0.001 is the significance of the difference between the control and the distinct treatments. (C) Proteolytic activity of live trichomonads over legumain substrate (Cbz-Ala-Ala-AAsn-AMC). Live parasites were incubated with the same inhibitors previously described in (A), and the released fluorescence from the legumain substrate was measured in a fluorometer. Each bar is the mean of the percentage of triplicate samples; error bars represent the standard deviations of three experiments in triplicate with similar results. *P < 0.001 is the significance of the difference between the control and the distinct treatments. (D) Proteolytic activity of live trichomonads over papain substrate (Z-Phe-Arg-AMC). Live parasites were incubated with the same inhibitors previously described in (A), and the released fluorescence from the papain substrate was measured in a fluorometer. Each bar is the mean of the percentage of triplicate samples; error bars represent the standard deviations of three experiments in triplicate with similar results. *P < 0.001 is the significance of the difference between the control and the distinct treatments.

To determine whether TvLEGU-1 proteolytic activity was required for cellular attachment, live nonradioactive-labeled parasites [41] were treated with distinct CP inhibitors (TLCK, leupeptin, or E-64) or with increasing concentrations (0, 5, 10, and 50 **μ**g/mL) of a specific legumain inhibitor the Aza-Peptidyl Michael Acceptor (Mu-Ala-Ala-AAsn-CH=CH–CON) [43] before interaction with live HeLa cell monolayers. Figures 8(A) and 8(B) show that the specific legumain inhibitor decreased the levels of *T. vaginalis *adherence to HeLa cell monolayers in a concentration-dependent manner up to ~80%, whereas TLCK, leupeptin, and E-64 inhibited ~60, ~40, and ~50%, respectively. The average number of parasites without treatment attached to HeLa cells per coverslip was higher than the number of parasites treated with inhibitors (Table 3), and these differences were statistically significant *P* < 0.001 (Figure 8(B)). These results suggest that both legumain and papain-like CP proteolytic activities are necessary for trichomonal cytoadherence, especially the legumain-like activity. These data are consistent with previous reports [4]. 

To check the effect of these inhibitors over the CP proteolytic activity of live parasites fluorescent substrates for papain-like (Z-Phe-Arg-AMC) and legumain-like (Cbz-Ala-Ala-AAsn-AMC) CPs were used. Live untreated parasites used as control showed proteolytic activity for both substrates, which were taken as 100% activity. Parasites treated with the specific legumain inhibitor (Aza-Peptidyl Michael Acceptor) abolished the legumain-like proteolytic activity (Figure 8(C)) and has no effect on the papain-like proteolytic activity (Figure 8(D)). TLCK, a potent inhibitor of papain-like and legumain-like CPs, greatly reduced both proteolytic activities (~80% and ~95%, resp.) of treated parasites as expected (Figures 8(C) and 8(D)). E-64 and leupeptin, potent inhibitors of papain-like CPs, greatly reduced the papain-like proteolytic activity (between ~80 to ~90%) of treated parasites (Figure 8(D)) and had a minimal or no effect on the legumain-like proteolytic activity (Figure 8(C)) of live parasites. Therefore, both types of CP proteolytic activity are present in live parasites and are necessary for trichomonal adherence to host cells (Figure 8).

### 3.7. The TvLEGU-1 Proteinase Is Expressed during Infection and Is Present in Vaginal Secretions of Patients with Trichomoniasis

**Figure 9:**
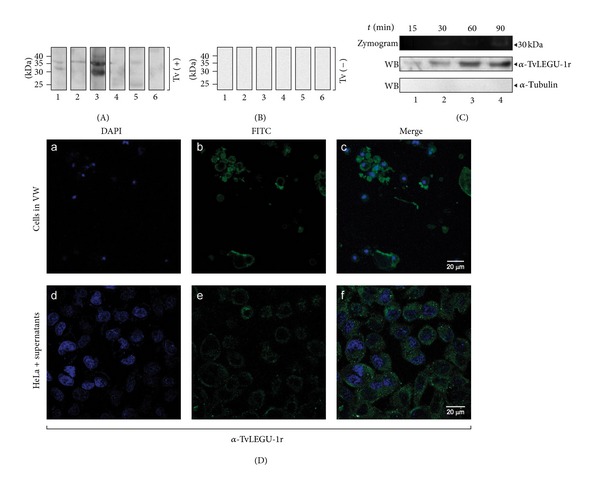
Presence of TvLEGU-1 in vaginal secretions and in *in vitro* secretion assays. (A) WB assays of TCA-precipitated proteins present in VWs from *T. vaginalis *positive culture patients [Tv (+)] (lanes 1–6) incubated with the anti-TvLEGU-1r antibody. (B) WB assays of TCA-precipitated proteins from people with other vaginitis [Tv (−)] used as negative controls (lanes 1–6) incubated with the anti-TvLEGU-1r antibody. (C) Zymogram and WB assays of the proteins present in the *in vitro *secretion products obtained from metabolically active parasites (1 × 10^6^ cells/mL) that were incubated in PBS-0.5% maltose at 37°C for 15, 30, 60, and 90 min (lanes 1–4, resp.). NC membranes containing the TCA-precipitated *in vitro *secretion products incubated with the anti-TvLEGU-1r antibody (1 : 10,000) or the anti-*α*-tubulin antibody (1 : 1000) used as a negative control. (D) Confocal microscopy images of fixed cells obtained from vaginal washes (VWs) and from live HeLa cell. (a, b, and c) Cells from VWs of patients with trichomoniasis confirmed by *in vitro* culture [Tv (+)] directly incubated with the anti-TvLEGU-1r antibody. (d, e, and f) Live HeLa cells incubated with supernatants from the *in vitro* secretion assays in which TvLEGU-1 is present (C) and with the anti-TvLEGU-1r antibody. Conjugated anti-rabbit IgG-FITC was used as a secondary antibody (1 : 100 dilution) (b and e). Nuclei labeled with DAPI (a and d);  merge, and bars: 20 *μ*m (c and f).

To investigate the relevance of TvLEGU-1 during trichomonal infection, we analyzed vaginal washes from vaginitis patients with [Tv (+)] or without [Tv (−)] *T. vaginalis* (Table 4) for the presence of TvLEGU-1 by TCA-precipitation and WB assays using the anti-TvLEGU-1r antibody.  Figure 9 shows that the anti-TvLEGU-1r antibody detected the presence of protein bands that ranged from 35- to ~30-kDa in Tv (+) VWs (Figure 9(A)), but none in those Tv (−) with other vaginitis (Figure 9(B)), used as negative controls. These data illustrate that the TvLEGU-1 is expressed and might be released during infection. 

To confirm this, *in vitro* secretion kinetic assays were performed (0, 15, 30, 60, and 90 min). Zymograms of the supernatants showed a 30-kDa band with proteolytic activity released through time (Figure 9(C)) from parasites that exhibited ~95 to ~99% viability measured by trypan blue exclusion assays. WB assays of the TCA-precipitated supernatants using the anti-TvLEGU-1r antibody confirmed the presence of TvLEGU-1 among the released proteins and its amount increased through time. The anti-*α*-tubulin antibody used as a negative control gave no reaction as expected, suggesting that no significant parasite lysis occurred. 

Moreover, immunofluorescence assays were also performed using the anti-TvLEGU-1r antibody with cells obtained from Tv (+) VWs and with live HeLa cells incubated with TvLEGU-1-containing parasite supernatants from the *in vitro* secretion assays (Figure 9(C)). The confocal microscopy images showed that endogenous TvLEGU-1 decorates the surface of live HeLa cells and cells obtained from Tv (+) VWs (Figure 9(D)). These data show that TvLEGU-1 is part of the excretion/secretion products from live trichomonads that also bound to the surface of cells present in vaginal secretions (Figure 9). 

## 4. Discussion

Numerous thiol-proteinases, including cathepsin L- and legumain-like proteinases, are encoded in the *T. vaginalis* genome. However, few have been characterized at either the molecular, biochemical, or functional level. Understanding the role of CPs, especially those of the legumain-like type in this parasite, is relevant, as they are known to be involved in numerous biological processes including the host-parasite interplay. 

In this study, we identified and characterized one of the ten legumain-like proteinases described in the draft of the *T. vaginalis* genome sequence [9], TvLEGU-1 [38], that showed multiple localizations, in the lysosomes and Golgi complex when in the cytoplasm and also at the parasite surface in the presence of iron (Figures 3–5). We confirmed its positive iron regulation [21] at the protein level (Figure 2) and surface localization (Figures 3 and 4). Moreover, we demonstrated its role in trichomonal adherence (Figures 7 and 8) and its presence in vaginal secretions during trichomonal infection (Figure 9). 

The three protein spots in the 30-kDa region, with distinct pI identified by 2DE WB and MS as TvLEGU-1 (Figure 1, Table 1), are in agreement with the protein spots identified as TvLEGU-1 in the trichomonad active degradome [15]. These represent isoforms with a different type and level of phosphorylation (Figure 1(D)) as previously suggested [38]. These findings are consistent with legumains from other organisms such as the Cs-legumain from *Clonorchis sinensis* with a similar pattern in 2DE gels [44], which is also phosphorylated. 

Interestingly, the anti-TvLEGU-1r antibody showed a different recognition in the WB assays depending of the protein preparation. This could be due to the presence of fewer amount of protein in the protease-rich extract corresponding to the different protein bands that the antibody was unable to detect them. However, previous data showed that a 60-kDa protein spot was also observed in the 2DE gels from protease-rich extracts, and it was identified as part of the TvLEGU-1 by MS [15]. At this point, we do not have an explanation to this high molecular size TvLEGU-1 protein. It is something that needs to be studied further. Although the 20-kDa protein was not observed nor identified by MS in the protease-rich extract 2DE gels [15], we can speculate that this protein could represent a processing stage of TvLEGU-1 or a degradation product. Additionally, we could not discard that these proteins are the intracellular forms of TvLEGU-1. It is also something that deserves further investigation to help to understand the way this protein is processed, activated, localized, and showed different functions that could be modulated by the iron concentrations in the microenvironment.

TvLEGU-1 is one of the 30-kDa CPs localized on the surface of iron-rich trichomonad parasites (Figure 3) that also bound to the surface of HeLa cells (Figures 6 and 9). These are properties consistent with the proteolytic activity necessary for trichomonal cytoadherence [4, 5, 7]. As expected, the antibodies against the recombinant TvLEGU-1r inhibited trichomonal adherence in a similar range (Figure 7) as the anti-CP30 antibody [7]. Additionally, *T. vaginalis *cytoadherence reduction by the specific legumain inhibitor supports that the TvLEGU-1 proteolytic activity may play a major role in the host-parasite interaction during trichomonal adherence (Figure 8). Therefore, this finding is consistent and corroborates our previous observations that the CP30 proteolytic activity is necessary for trichomonal adherence [4, 5, 7] and a legumain-like CP, TvLEGU-1, is part of it. Additionally, TvLEGU-1 is positively regulated by iron at the protein level (Figure 2) and surface localization (Figures 3 and 4), similar to the iron upregulation of the trichomonad adhesins [6, 41, 42, 45]. This behavior could be expected for molecules that participate in the same trichomonal virulence property, cytoadherence [4, 6, 7, 45]. Interestingly, the lack of surface recognition of the anti-TvLEGU-1r antibody in the Triton X-100-permeabilized parasites could be explained based on previous reports. These demonstrate that nonionic detergents, such as Triton X-100, redistribute and solubilize phospholipid anchored proteins, even in previously fixed cells. Detergents such as Triton X-100 also have significant adverse affects on the immunochemical analysis of gangliosides and GPI-anchored proteins [46, 47]. Consistent with this explanation recent unpublished data suggest that TvLEGU-1, lacking transmembrane domains [38], is on the parasite membrane through a putative phospholipid anchor (work in progress).

The localization of the TvLEGU-1 in different compartments, particularly in lysosomes, suggests the typical role for TvLEGU-1, participating in the lysosomal degradation of food [48–50] or internal organelles during an autophagy process for remodelling of the cellular components [51] or in the parasite metabolism. This is in addition to its new role as a virulence factor involved in cytoadherence, as has been demonstrated in here, supporting previous data [6, 41, 42]. Moreover, the localization of TvLEGU-1 in the Golgi complex suggests that this CP undergoes part of its processing and maturation steps in this organelle as occurs with other legumains. Commonly, these proteinases are translated as a preproform, transferred through the Golgi complex as the proform of legumain with a molecular mass of 56-kDa, and localized in late endocytic compartments as the mature enzyme with a molecular mass of 46-kDa [52], as occurs in lysosomal cysteine proteinase [53].

The legumain CPs, which belong to the clan CD, are distinct from those of all other clans (with regards to their amino acid sequence, tertiary structure fold, protein substrates, effect of inhibitors, and biological functions). Their discovery has led to a reassessment of the relevance of roles played by CPs in parasitic protozoa [23, 24]. Moreover, legumains have greater specificity in their functions than the clan CA enzymes. Thus, the fact that legumain-like CPs have a very restricted type of substrates [48], as compared with the papain-like CPs, which are very promiscuous [23, 24], suggests that this could be one of the major proteolytic activities necessary for trichomonal adherence to host cells [4, 5, 7]. We can speculate that this type of CPs such as TvLEGU-1 will unmask the surface of *T. vaginalis *by degrading the host proteins covering the adhesins, as previously suggested [4, 5, 54]. However, it is important to mention that as shown here the proteolytic activity of CPs of both clans, CA and CD, is necessary for trichomonal cytoadherence (Figure 8). One explanation is that both types of CPs could be necessary for directly processing the same protein targets such as the host proteins that cover the trichomonad surface [4, 5, 54]. Another possible explanation is through a similar mechanism as the one described for hemoglobinolysis of parasitic organisms such as apicomplexan and nematodes, where the participation of several proteinases is necessary in a cascade of proteolytic activation. A clan CD cysteine peptidase of the legumain type is implicated in the first step activating proteinases of clan CA directly involved in hemoglobin degradation [55–57]. 

Therefore, we could speculate that in *T. vaginalis *an activation cascade could also be occurring to uncover the parasite surface, in which TvLEGU-1 will participate in the first step activating the papain-like CPs involved in host protein degradation. Once trichomonad CPs digest the proteinaceous cover on the *T. vaginalis* surface, by any of the two proposed pathways, the adhesins will then be free to interact with the host cell receptors for attachment [6, 41, 42]. Thus, further studies will be required to determine whether TvLEGU-1 directly participates in host protein degradation or in the first step of a putative activation pathway that will degrade particular host proteins as a prerequisite for cytoadherence. It also will be relevant to identify the substrate proteins for TvLEGU-1. Further work is needed to examine these questions.

It is relevant to mention that the recombinant protein TvLEGU-1r obtained in this study did not have proteolytic activity and could not be activated (data not shown) for biochemical and direct functional assays, using the same reported conditions [58]. This could be due to the fact that the *tvlegu-1 *gene was expressed in bacteria and lacked the sequence coding the first 10 aa residues of the N-terminal region, which may be required for its correct folding and activation. In spite of that, the recombinant TvLEGU-1 interacted with fixed HeLa cell, as the native protein did, suggesting that, in TvLEGU-1, the cell-binding and catalytic domains are different and could function separately. Moreover, identification of TvLEGU-1 in vaginal washes from women with active trichomoniasis is consistent with the presence of the CP30 proteolytic activity [7] in trichomoniasis symptomatic patients [7, 59], suggesting that, during infection, *T. vaginalis* releases several CPs, including TvLEGU-1, which is highly immunogenic [15]. Interestingly, we observed several bands (35- to ~30-kDa) specifically recognized by the anti-TvLEGU-1r antibody in the Tv (+) VWs analyzed, suggesting the presence of different processing states of TvLEGU-1 during infection. Further work is needed to determine the processing steps of TvLEGU-1.

## 5. Conclusion

In this work, we have demonstrated that indeed CP proteolytic activity is necessary for trichomonal adherence to host epithelial cells that is consistent and corroborates our previous observations. One of these CPs is the TvLEGU-1, a legumain-like CP that is located in lysosomes, Golgi complex, and at the parasite surface in the presence of iron and shows different levels of phosphorylation. It will be interesting to identify the particular substrates for this CP, in addition to determine the phosphorylation or dephosphorylation effect on the proteolytic activity and its impact on cytoadherence. This CP was also found in vaginal secretions of patients with trichomoniasis, supporting its potential as biomarker. This work is the first paper that shows that a legumain-like CP plays a role in a pathogen cytoadherence. 

## Supplementary Material

Figure 1S. Matched peptides identified in the three protein spots recognized by the anti-TvLEGU-1r antibody in the deduced amino acid sequence of the TvLEGU-1 protein. Boxes in gray show the matched peptides obtained from tryptic digestion and mass spectrometry (Table 1). Consecutive Roman numbers (I-X) were assigned to the identified peptides.

## Figures and Tables

**Table 1:** Peptides identified by MALDI-TOF-MS analysis of the three TvLEGU-1 protein spots of* Trichomonas vaginalis *detected by the anti-TvLEGU-1r antibody*. *

Peptide number^a^	Position^b^ (aa)	Number in the sequence^c^	*m*/*z* (av)^d^	Spot 1^e^	Spot 2^e^	Spot 3^e^	Amino acid sequence^f^
1	13–27	I	1749.9186	−	+	+	FAVLIAGSNDFYNYR
2	28–41	II	1734.9719	+	+	+	HQADIFNMYQQLVK
3	41–66	III	2774.0025	−	+	+	GFDDQHITMMAYDDIALSSENPFR
4	42–66	IV	2930.1882	−	+	+	RGFDDQHITMMAYDDIALSSENPFR
5	75–84	V	1101.2126	−	+	+	HVNIYPGSSK
6	85–105	VI	2399.6528	−	+	+	INYAHNSVTADQFYTVLTTLK
7	144–151	VII	911.4059	+	+	−	AFDTMEAK
8	157–174	VIII	1994.1426	−	+	+	LFFGIEACYSGSVAAVFR
9	157–176	IX	2193.5226	+	+	+	LFFGIEACYSGSVAAVFRAK
10	232–248	X	1931.1107	−	+	+	AQTTGSHVCYYGDVNMK

^a^Consecutive number assigned to the identified peptides. ^b^Position in amino acids (aa) residues of the identified peptides (start-end) in the aa sequence of the *T. vaginalis* TvLEGU-1 [38]. ^c^Arbitrary nomenclature used to describe the ten identified peptides in *T. vaginalis* TvLEGU-1 (see Supplementary Figure  1S in Supplementary Material available online at doi:10.1155/2012/561979). ^d^Peptide mass average *m*/*z* (av) identified by MALDI-TOF-MS after tryptic digestion of the three protein spots obtained from 2DE of protease-rich extracts from *T. vaginalis* grown in normal iron conditions (Figure 1). ^e^Presence (+) or absence (−) of the peptides identified by MALDI-TOF-MS in the three TvLEGU-1 protein spots analyzed. ^f^Amino acid sequence of the peptides obtained from a theoretical tryptic digestion of the deduced aa sequence of TvLEGU-1 [38] with identical masses to the experimental one (Supplementary Figure  1S).

**Table 2:** Densitometric analysis of the three TvLEGU-1 protein spots observed in silver-stained gels and WB NC membranes from parasites grown in high and low iron concentrations.

Spot number	Pixel intensity of silver stained spots		Pixel intensity of WB spots	
	High iron^a^	Low iron^b^	High iron^c^	Low iron^d^
1	292685.95	83473.87	93367.94	29862.21
2	637882.72	416358.36	296207.75	245016.26
3	1781948.40	1171066.01	842341.15	794637.06

^a^Densitometry values of the spots detected in the silver stained gel in high iron condition (H) of Figure 2(A). ^b^Densitometry values of the spots detected in the silver stained gel in low iron condition (L) of Figure 2(A). ^c^Densitometry values of the spots detected in the WB NC membranes in high iron condition (H) of Figure 2(A). ^d^Densitometry values of the spots detected in the WB NC membranes in low iron condition (L) of Figure 2(A).

**Table 3:** The average number of *T. vaginalis *parasites attached to HeLa cell monolayers per coverslip with or without treatment using different proteinase inhibitors (Figure 8(B)).

Treatment	Parasites attached	Inhibition (%)
None^a^	1450	0.0 ± 13.9176
MA (5 *μ*M)^b^	742	46.33 ± 7.7764
MA (10 *μ*M)	556	60.78 ± 5.6311
MA (50 *μ*M)	301	77.23 ± 5.2907
TLCK (1 mM)	622	57.05 ± 6.6416
Leupeptin (0.2 mM)	898	36.79 ± 6.1359
E-64 (0.18 mM)	662	50.16 ± 8.1047

^a^None corresponds to the control parasites without treatment with CP inhibitors. The number of parasites attached to the HeLa cell monolayer was taken as 100% adherence for comparative purpose. ^b^MA corresponds to the Aza-Peptidyl Michael Acceptor, a legumain-specific inhibitor. These differences were statistically significant with a *P* < 0.001 (Figure 8(B)).

**Table 4:** Characteristics of the biological samples from patients with vaginitis.

Sample number^a^	Clinical diagnosis^b^	*T. vaginalis* ^ c^		Other microorganisms^d^
		Wet mount	InPouchTv
HGM483	+	−	+	*Corynebacterium *sp., coagulase-negative* Staphylococcus *sp*. *
HGM315	+	+	+	*Lactobacillus *sp., coagulase-negative* Staphylococcus *sp*. *
HGM295	+	+	+	+^e^
HGM225	+	+	+	Coagulase-negative* Staphylococcus *sp*. *
HGM124	+	+	+	*Corynebacterium *sp.
HGM114	+	+	+	+^e^
HGM9	−	−	−	*Candida *sp*. *
HGM39	−	−	−	*Corynebacterium *sp*. *
HGM47	−	−	−	*Candida *sp.
HGM48	−	−	−	*Corynebacterium *sp*. *
HGM67	−	−	−	*Candida *sp.*, Corynebacterium *sp*. *
HGM331	−	−	−	Coagulase-negative* Staphylococcus *sp*., Gardnerella vaginalis, Lactobacillus *sp*. *

^a^Biological samples obtained from Laboratorio Central del Hospital General de México (HGM). ^b^Clinical diagnosis of cervicovaginitis in all patients. ^c^Presence of *T. vaginalis* detected by direct wet mount microscopic observation and by *in vitro* culture with the InPouchTv system. ^d^Presence of bacteria in the wet mount that were identified by a microbiological analysis. ^e^Presence of bacteria in the wet mount that were not identified by a microbiological analysis as the rest of the samples.
