# Supplementary material for: The TvLEGU-1, a Legumain-Like Cysteine Proteinase, Plays a Key Role in Trichomonas vaginalis Cytoadherence
Source: Biomed Res Int. 2013 Jan 1;2013:561979. doi: 10.1155/2013/561979 (PMC3581150; doi:10.1155/2013/561979)
Supplement: Supplementary file 1 — Figure 1S. Matched peptides identified in the three protein spots recognized by the anti-TvLEGU-1r antibody in the deduced amino acid sequence of the TvLEGU-1 protein. Boxes in gray show the matched peptides obtained from tryptic digestion and mass spectrometry (Table 1). Consecutive Roman numbers (I-X) were assigned to the identified peptides. [file 561979.f1.pdf]

Figure 1S

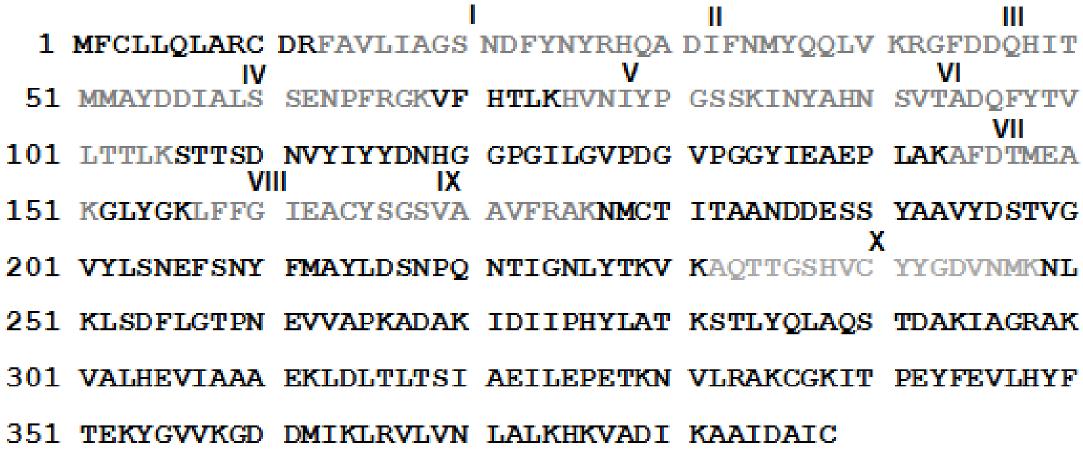

Supplementary figure legend

Figure 1S. Matched peptides identified in the three protein spots recognized by the anti-TvLEGU-1r antibody in the deduced amino acid sequence of the TvLEGU-1 protein. Boxes in gray show the matched peptides obtained from tryptic digestion and mass spectrometry (Table 1). Consecutive Roman numbers (I-X) were assigned to the identified peptides.
